# From Episodic Checks to Continuous Clinical Monitoring: Nursing Policy Viewpoint on AI-Enabled Oral and Nutrition Risk Detection in Long-Term Care

**DOI:** 10.2196/87598

**Published:** 2026-10-05

**Authors:** Miya Aishima, Kazumi Kubota

**Affiliations:** 1 Department of Nursing, Shimonoseki City University, Shimonoseki, Yamaguchi, Japan; 2 Research Organization, Shimonoseki City University, 2-1-1 Daigaku-cho, Shimonoseki, Yamaguchi, 751-8510, Japan, 81 83-252-0288; 3 Department of Healthcare Information Management, University of Tokyo Hospital, Tokyo, Tokyo, Japan; 4 Division of Women's Health Promotion Research, Department of Women's Health Population Sciences, National Center for Child Health and Development, Tokyo, Tokyo, Japan

**Keywords:** long-term care, nursing, artificial intelligence, AI, malnutrition, oral health

## Abstract

Oral disease and malnutrition are common and closely linked problems in long-term care (LTC). Monthly weights, occasional diet reviews, and infrequent dental assessments can miss gradual decline. Recent tools, including computer-vision meal-intake estimation and smartphone-based gingival screening, create an opportunity for more timely clinical monitoring when limited data capture is embedded into routine care. This viewpoint proposes a nursing-led policy framework for AI-enabled oral and nutrition risk detection in nursing homes. The framework emphasizes protected oral health and nutrition champion roles, a practical 48-hour bedside assessment standard for high-priority operational alerts, standards-based electronic health record (EHR) integration, and prevention-oriented escalation pathways. We clarify that the proposed approach does not require a single black-box AI risk score. Instead, AI-derived measurements, such as estimated intake, plate-waste ratio, deviation from baseline, and image-based oral findings, can be combined with weight trends, EHR data, operational thresholds, and nurse review. The playbook specifies staged rollout, staff-facing alert outputs, fidelity checks for data capture, fallback documentation options for facilities with lower digital maturity, and key performance indicators for clinical outcomes, workflow burden, equity, and cost. Ethical safeguards include layered consent, minimum-necessary capture, opt-out recording, explainability for residents and proxies, and subgroup monitoring. AI-enabled clinical monitoring can support earlier action in LTC only if it is embedded in nursing workflows, auditable documentation, and accountable governance. Prospective, co-designed implementation studies are needed to test feasibility, workload, effectiveness, and equity across diverse LTC settings.

## Introduction

In many nursing homes, oral discomfort and inadequate intake accumulate quietly. By the time weight loss is visible or infection is diagnosed, small preventive actions have been missed. Such missed opportunities indicate areas where long-term care (LTC) governance could be strengthened. This paper focuses on facility-based LTC rather than assisted living or home care, because staffing patterns, shared dining, and documentation practices in nursing homes create specific opportunities and constraints for clinical monitoring and intervention. When we use the term “clinical surveillance,” we mean care-oriented risk monitoring based on minimum-necessary data capture, not continuous visual observation of residents or monitoring of behavior for noncare purposes. The goal is to notice potentially reversible intake decline or oral discomfort earlier while preserving resident dignity, privacy, and relational care. Feasibility studies show that image-based intake tracking, such as automated food imaging and nutrient intake tracking, estimates nutrient consumption with good agreement to manual methods in LTC environments [1]. Mobile intraoral imaging tools, including generative AI, have detected gingival inflammation with high sensitivity among older adults [2]. These innovations address a persistent unmet need. Cross-sectional and cohort studies report poor oral hygiene, chewing difficulties, and substantial untreated disease among residents [3], while structured instruments underdetect oral problems unless protocols are strengthened [4].

Even well-designed implementation strategies often fail to convert staff knowledge into measurable oral-health gains [5,6]. Parallel work suggests that malnutrition risk can be signaled before overt clinical decline by combining weight trajectories, appetite changes, and other EHR features [7,8]. Ethical and governance literature emphasizes transparency, explainability, and accountability for clinical AI [9]; nursing scholarship calls for new competencies and roles to support a digital future [10,11]; and oral-health equity discussions warn against repeating historical disparities [12]. Social and behavioral perspectives remind us that oral care in older adults is not only clinical but also relational [13].

From an informatics standpoint, standards-based integration with the electronic health record (EHR) is a precondition for sustainability; Substitutable Medical Applications and Reusable Technologies (SMART) on Fast Healthcare Interoperability Resources (FHIR) offers a practical route to bidirectional exchange without bespoke interfaces [14]. Decision-support research shows that poorly calibrated alerts erode trust [15], and implementation science supplies concepts and measures for adoption, fidelity, and sustainability [16]. The growing burden of noncommunicable disease in aging populations [17,18] strengthens the case for prevention-oriented, nursing-led digital clinical monitoring, aligned with the evolution of nursing informatics [19] and consistent with international guidance on AI ethics in health [20]. The sections that follow synthesize this evidence into a policy framework and a pragmatic playbook tailored to nursing homes.

## Evidence Base and Conceptual Approach

This viewpoint synthesizes published evidence and implementation concepts; it involves no new data or human participants. The review privileges studies of computer-vision intake tracking in LTC [1], mobile intraoral screening in older adults [2], and investigations into oral-health burden and underdetection in nursing homes [3-6], along with AI-based malnutrition risk modeling [7,8]. It also draws on scholarship in ethics and equity for healthcare AI [9,12,20], nursing’s digital transformation [10,11,19], clinical decision support and alert fatigue [15], interoperability standards [14], and implementation outcomes [16]. Conceptually, the synthesis was informed by implementation science, particularly the framework of adoption, fidelity, and sustainability proposed by Proctor et al [16]. Ethics approval is not applicable.

## Proposed Framework and Operational Specification

Subsequent sections present the proposed policy framework and the operational details intended to make it usable at the bedside.

### Nursing-Led Policy Framework

The framework rests on 5 elements that reinforce one another and can be audited in everyday work. These elements were conceptually informed by prior implementation and intervention evidence, interpreted through nursing policy priorities of (1) governance, (2) timeliness, (3) integration, (4) ethics, and (5) incentives.

Governance begins with the designation of 2 nurse champions, 1 for oral health and 1 for nutrition. Their remit is to review alerts, coordinate bedside assessments, oversee documentation and follow-up, and convene a weekly huddle to monitor performance. A planning assumption of 0.2 to 0.4 full-time equivalent per 100 beds is offered as a practical staffing starting point to help ensure that this work is not squeezed by routine duties. This estimate was not derived from a formal Delphi consensus, historical workload dataset, or predictive staffing model. Rather, it is an author-proposed planning assumption informed by clinical and implementation experience and based on the expected tasks of alert review, staff support, documentation oversight, education, troubleshooting, and interdisciplinary coordination during pilot implementation. It should therefore be treated as a local design hypothesis to be tested and calibrated, not as a validated universal benchmark [10,11,19]. Timeliness is formalized through a 48-hour response standard: any high-priority operational alert for suspected oral or nutrition decline triggers bedside assessment within 48 hours. This threshold functions as a practical floor that matches typical LTC operational cycles and the time sensitivity of oral pain and intake decline. Similar to the staffing estimate, it was not derived from a formal consensus, historical outcome modeling, or a validated clinical threshold. It is an author-proposed operational standard informed by clinical and implementation experience, intended for pilot implementation and local calibration according to staffing patterns, specialist availability, resident acuity, and organizational context.

Where staffing or specialist access requires it, facilities may use the variant “within 48 hours or the next business day,” while preserving the same spirit of urgency. Integration reduces duplication and prevents missed follow-ups. Alerts and actions are written back to the EHR using SMART on FHIR and common terminologies so that bedside work becomes part of the resident’s record and is visible to the entire team [14]. Ethical safeguards include layered consent, opt-out recording, and minimum-necessary capture. Residents and proxies receive clear explanations of what information is collected, why it is useful, and how it is protected. Subgroup performance is monitored to detect inequities, following guidance on responsible AI in oral health and broader ethical frameworks [9,12,20]. Finally, incentives should reward prevention. Adherence to the 48-hour rule and measurable improvements (eg, fewer unplanned transfers or stabilization of weight) can be linked to quality bonuses or prevention-oriented reimbursement. Implementation outcomes provide the vocabulary and structure for consistent reporting across sites [16].

### EHR Integration Specifics

While integration across heterogeneous EHR systems remains challenging, a small set of structured data flows is sufficient to move from demonstration to sustainable practice. Observations, either automatically generated or nurse-verified, capture component-level measurements such as plate-waste estimates, estimated intake, operational alert status, and weights with appropriate Logical Observation Identifiers Names and Codes and provenance. Conditions record referable oral findings, including gingivitis and denture-related lesions, in Systematized Nomenclature of Medicine Clinical Terms. ServiceRequest entries initiate dentistry, dental hygiene, or dietetics consults when indicated. Tasks assign and time stamp the 48-hour bedside assessment so that the interval from alert to assessment can be audited. CarePlans, when used, record short-term goals and interventions such as fortified diets or oral-care regimens. AuditEvent and Provenance resources document model versions and threshold settings so that performance can be traced over time [14].

For facilities with lower digital maturity or legacy EHRs that are not FHIR-native, a transitional approach may be necessary. Such facilities can begin with a minimal structured dataset, standardized documentation templates, controlled terminology where feasible, CSV or batch export from the application, and scheduled reconciliation into the EHR by designated staff. This fallback should be treated as a temporary maturity step rather than a permanent parallel workflow, as duplicate documentation and spreadsheets can increase workload and weaken auditability. Any fallback workflow should have a named owner, a defined reconciliation schedule, and a sunset criterion for transition toward structured EHR integration. The implementation goal should remain focused on progressive movement toward structured EHR integration and, where feasible, standards-based write-back.

### Consent and Privacy Safeguards

Ethics become concrete, particularly in practice, when imaging occurs in shared dining areas and when many residents live with cognitive impairment, both situations grounded in nursing ethics of relational autonomy and beneficence. Layered consent accommodates a facility-level policy notice and posted signage, intake or annual consent that may involve proxies, and brief reminders at the bedside when images are captured. Minimum-necessary capture focuses images on plates and mouths, avoids faces and bystanders, and favors on-device processing with automatic masking. Data should be stored in secure, access-controlled systems using encryption in transit and at rest, with role-based access logging and retention periods limited to the minimum necessary for clinical care, quality assurance, and audit. Facilities should define local protocols for review, retention, secure deletion, or irreversible anonymization once identifiable data are no longer required, in alignment with applicable legal and institutional requirements and with established information-governance frameworks such as General Data Protection Regulation principles and ISO 27001–based controls. Opt-out status is recorded in the EHR and respected by default. Quarterly reviews examine subgroup performance to detect bias or drift and, where appropriate, incorporate structured input from residents, proxies, family representatives, or advocacy groups regarding acceptability, perceived intrusiveness, and relational aspects of care, drawing on equity-focused guidance for oral-health AI and international frameworks for AI in health [12,20].

Figure 1 provides a visual summary of the 2-stage workflow, illustrating how routine or semiroutine monitoring supports early signal detection and operational alerts, how selective intraoral imaging is used as a follow-up step when indicated, and how KPI (key performance indicator) feedback informs threshold tuning.

**Figure 1.**
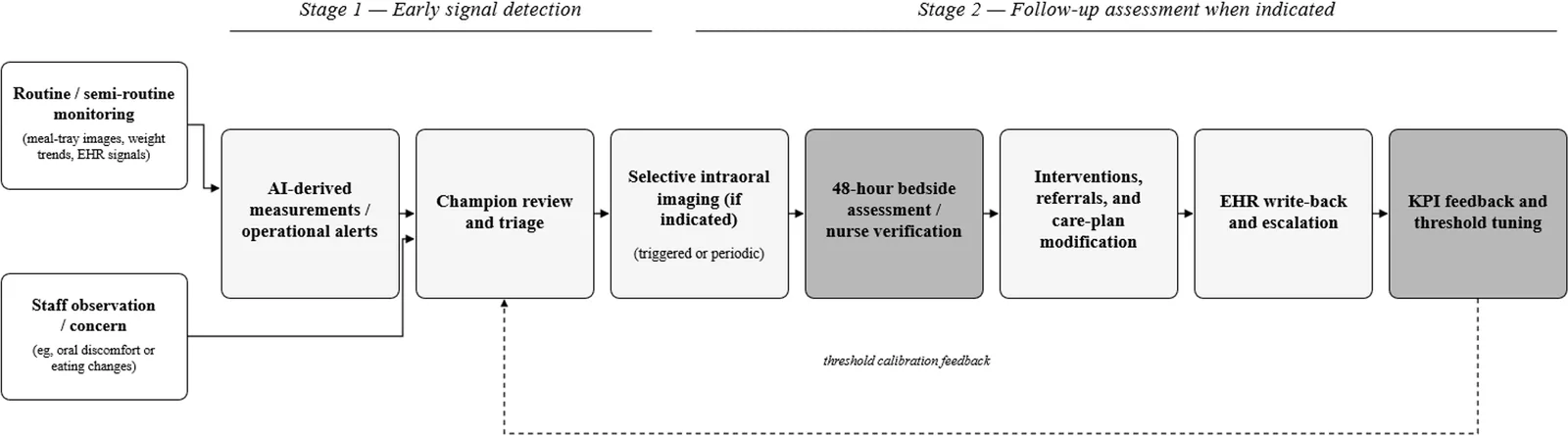
Two-stage workflow for AI-enabled oral and nutrition clinical monitoring in long-term care. EHR: electronic health record; KPI: key performance indicator.

### Implementation Playbook

In practical terms, the proposed intervention is a 2-stage nursing workflow rather than a single black-box AI risk score, as presented in Figure 1. First, routine or semiroutine data streams are used to detect early signals of change. These include meal-tray images captured at selected time points, weight trends, and relevant EHR information such as appetite notes, dysphagia, denture problems, or recent infections. AI-enabled intake tools may provide component-level measurements, such as estimated intake, plate-waste ratio, or percentage deviation from a resident’s usual intake.

Second, when these measurements cross predefined operational thresholds, or when staff identify oral discomfort or eating changes, selective intraoral imaging can be used to clarify whether oral factors may be contributing. In this role, oral imaging supports confirmation, severity description, standardized documentation, and referral decisions within the validated scope of the tool; it is not presented as the sole early-detection mechanism. Facilities may choose 1 of 2 oral-imaging modes depending on capacity: a triggered mode, in which intraoral images are obtained after intake decline, chewing discomfort, or staff concern; or a limited periodic mode, such as weekly or monthly imaging for residents at higher baseline risk. The framework does not assume that all residents receive daily oral imaging.

Staff-facing outputs should be specific and interpretable. For example, an alert may state that “estimated intake has fallen by approximately 30% from this resident’s two-week baseline over five days,” rather than simply displaying a global “high-risk” label. If oral imaging is performed, a separate output may indicate “possible gingival inflammation” or “possible denture-related lesion,” prompting nurse verification. Facilities may combine these component outputs with weight trends, EHR data, and nurse judgment using locally defined rules. Thus, the alert is an operational trigger for bedside review, not necessarily the direct output of a trained AI risk-prediction model. Image capture is performed by nursing staff or designated caregivers, while nurse champions oversee alert review, workflow coordination, documentation, and threshold calibration.

Whereas Table 1 outlines policy levers and KPIs, Table 2 specifies the minimum bedside workflow and fidelity checks needed to interpret these KPIs during pilot implementation.

**Table 1.** Policy levers, operational actions, and key performance indicator (KPI) definitions for AI-enabled oral and nutrition clinical monitoring in long-term care^a^.

Policy lever	Operational action (facility level)	KPI and operational definition	Primary data source (EHR^b^ or app)
Governance and roles	Designate oral-health and nutrition nurse champions with protected time and run weekly alert huddles	Champion coverage: full-time equivalent per 100 beds (planned vs actual)	HR^c^ roster; meeting minutes
Timely response	Apply a 48-hour bedside assessment standard for high-priority operational alerts and document escalation	Alert-to-assessment time: percentage of high-priority operational alerts with documented bedside assessment within 48 hours; median alert-to-assessment time	Task time stamps in EHR
Integration and documentation	Write alerts, assessment outcomes, and follow-up actions back to the EHR via SMART^d^ on FHIR^e^ and avoid parallel spreadsheets	Documentation completeness: percentage of assessed alerts with structured EHR write-back of assessment outcome and follow-up action within the observation window	ServiceRequest; CarePlan; progress notes
Alert follow-up	Review flagged alerts for documented intervention, referral, or care-plan modification	Action rate: percentage of reviewed alerts that result in a documented intervention, referral, or care-plan modification within the observation window	Progress notes; ServiceRequest; CarePlan
Oral-care interventions	Implement SOPs^f^ for hygiene, denture checks, and pain control	Oral-care intervention rate: number of documented oral-care interventions per 100 resident-days during the observation window	Nursing oral-care charting; CarePlan
Nutrition interventions	Diet modification, fortification, supplements, and dietitian referral	Change in plate-waste ratio from baseline (percentage points)	Intake app Observation; EHR nutrition flowsheets
Outcomes-transfers	Track unplanned ED^g^ or hospital transfers monthly	Unplanned transfer rate per 100 resident-months during the observation window	Admissions or transfer logs
Outcomes-weight	Monitor monthly weights and trends	Weight-loss incidence: percentage of residents with ≥5% weight loss over 30 days	Observation (weight) with LOINC^h^
Staff burden	Measure documentation time for oral or nutrition tasks	Mean documentation time per shift (EHR logs or time-sampling)	Time-motion sample or EHR activity logs
Equity and bias	Conduct quarterly subgroup performance review (denture status, dysphagia, cognitive status, sex, and race or ethnicity where available)	Subgroup performance review: action rate and timely-response metrics by subgroup; gaps ≤predefined margin	EHR demographics; alert logs
Learning and calibration	Tune thresholds every 2‐4 weeks during the pilot, then monthly; record model version and rationale	Proportion of alerts reviewed at tuning meeting; presence of versioned change log	AuditEvent or Provenance; meeting records

a All key performance indicators (KPIs) should be reported with a numerator, denominator, and observation window. Some indicators, such as action rate, may require periodic manual review during the pilot phase. KPIs reflect core implementation outcomes, including adoption, fidelity, feasibility, and sustainability [16].

b EHR: electronic health record.

c HR: human resources.

d SMART: Substitutable Medical Applications and Reusable Technologies.

e FHIR: Fast Healthcare Interoperability Resources.

f SOP: standard operating procedure.

g ED: emergency department.

h LOINC: Logical Observation Identifiers Names and Codes.

**Table 2.** Minimum workflow specification, AI outputs, fidelity risks, and fallback options for pilot implementation.

Workflow component	Main purpose	Staff-facing output	Key fidelity risks	Pilot fidelity measures	Transitional option for lower digital maturity
Meal-tray image capture	Detect intake decline using routine or semiroutine mealtime data	Estimated intake, plate-waste ratio, and percentage deviation from resident baseline	Missed images, residents sharing food, spills, tray clearing in batches, and difficulty linking tray to resident	Image completion rate, image quality score, tray-resident linkage errors, reasons for missed capture, and time per capture	Paper or tablet checklist with standardized meal categories; batch CSV export from app where available
Weight and EHR review	Add clinical context to intake signals	Weight change, appetite notes, dysphagia, denture issues, and infection or medication changes	Delayed weight entry, free-text documentation, and inconsistent terminology	Completeness of weight records and proportion of alerts with relevant EHR^a^ context reviewed	Minimal structured template in EHR or secure spreadsheet during pilot
Operational alert generation	Convert measurements into a bedside review trigger	Plain-language alert, eg, “intake down approximately 30% from baseline over five days”	Overalerting, underalerting, unclear thresholds, and staff distrust	Alerts per nurse per week, false-positive review, and missed or delayed assessments	Rule-based thresholds reviewed in weekly huddles before automated integration
Selective intraoral imaging	Clarify whether oral factors may explain intake decline or discomfort	Image-based finding, such as possible gingival inflammation or denture-related concern, and requiring nurse verification	Poor lighting, resident discomfort, limited cooperation, and unclear field of view	Image quality, proportion of indicated images completed, and resident refusal or opt-out rate	Structured oral assessment template with optional photo attachment if permitted
Nurse-led bedside assessment	Translate alert into clinical action	Assessment outcome, intervention, referral, or care-plan modification	Competing workload, delayed assessment, and low-value interventions	Alert-to-assessment time, action rate, staff time per assessment, and missed assessment rate	Designated weekly review list with manual EHR entry
EHR write-back and audit	Keep alert, action, and outcome visible to care team	Structured note, Task, ServiceRequest, CarePlan, and AuditEvent or Provenance where available	Legacy EHR limits, duplicate documentation, and incomplete audit trail	Documentation completeness, duplicate-entry burden, and presence of versioned change log	Standardized template, CSV import or export, scheduled reconciliation, with transition plan toward FHIR^b^-based integration

a EHR: electronic health record.

b FHIR: Fast Healthcare Interoperability Resources.

A practical path begins with a unit-level pilot lasting 8 to 12 weeks. Before the start date, teams assemble baseline measures of unplanned transfers per 100 resident-months, weight-loss prevalence, oral-care intervention counts, “plate-waste” ratios, and staff documentation time per shift. Short, role-targeted training covers standardized intraoral photography, interpretation of AI-derived measurements and operational alerts, consent scripts, and EHR documentation. Selection of AI systems for intake estimation or oral-health assessment should consider procurement-relevant criteria, including auditability of model outputs, traceability of model versions and updates, documentation of training and validation data provenance, mechanisms for human review and override, and the vendor’s approach to postdeployment monitoring and change management. During the pilot, the champions chair a weekly huddle to examine false positives, near misses, and the interval from alert to assessment. Thresholds are tuned iteratively to balance sensitivity, actionability, and workload, with alert volume reviewed explicitly during weekly huddles [15]. After approximately 3 months, leaders decide whether to extend to other units or shifts, repeat training, or pause for further adjustment. Previous studies of AI-supported systems in LTC have shown that operational parameters such as alert thresholds and response timelines require local calibration depending on workflow and organizational context, reflecting practical implementation challenges such as alert burden, workflow integration, and staff workload, which have been identified as key barriers in prior pilot and implementation studies [21,22]. A lightweight dashboard reports 3 sentinel indicators each week—alerts per nurse, the proportion of alerts that lead to action, and the median alert-to-assessment interval. These indicators were selected as pragmatic pilot measures intended to balance timely response, actionability, and manageable alert burden in routine workflows, consistent with prior decision-support and implementation literature [15,16,21,22]. Some indicators (eg, action rate) are not part of routine frontline tasks and are instead assessed separately as part of quality improvement activities.

As starting points rather than mandates, facilities may begin by monitoring whether alert volume remains in an illustrative range, such as 5 to 10 alerts per nurse per week, while recognizing that acceptable volume will vary by staffing, resident acuity, shift patterns, and local workflow. Facilities may also track an action rate of approximately 30% and a median alert-to-assessment interval of approximately 24 hours, while retaining 48 hours as the outer operational limit for high-priority operational alerts. These values are intended as pilot-stage benchmarks for local calibration rather than validated universal performance standards. The definitions for these indicators, together with related outcome measures and data sources, are consolidated in Table 1. These indicators correspond to implementation outcomes of adoption, fidelity, and feasibility [16], allowing cross-site comparison. Importantly, this approach does not imply blanket increases in comprehensive manual assessment compared with conventional episodic checks; instead, it embeds limited data capture into routine care and uses AI-assisted processing to support more continuous signal detection, with staff responses triggered only when predefined operational thresholds are met. Table 1 summarizes policy levers, operational actions, and KPI definitions, providing a reference for auditing and reporting.

To ensure feasibility in routine LTC practice, the proposed system is designed to align with existing care workflows and minimize additional staff burden, while providing a structured pathway for implementation and evaluation. Meal intake image capture introduces a small additional task; however, it can be kept brief and operationally feasible by integrating it into routine meal-related activities and distributing responsibility across a limited number of staff (eg, a small number of staff per shift or a rotating group). Image capture is strategically limited to key time points—typically immediately before meal delivery (to document portion size, which may vary across residents) and at tray collection (to assess remaining intake while the tray can still be reliably linked to the resident). Capturing a single meal-tray image may take only a few seconds under ideal conditions, but mealtime in understaffed nursing homes is often crowded and time-pressured. Residents may share food, spill food or beverages, receive assistance from multiple staff members, or have trays cleared in batches. These behaviors and environmental conditions can affect image completeness, image quality, and accurate resident-tray linkage. Pilot implementation should therefore measure image completion rates, reasons for missed capture, image quality, linkage errors, and staff time, rather than assuming perfect fidelity. Intraoral image acquisition is not performed on a daily basis but is implemented selectively to maintain feasibility. In practice, images are obtained at limited intervals (eg, approximately once weekly) or in response to clinically relevant changes, such as reduced food intake, signs of oral discomfort, or observed alterations in eating behavior. Image capture can be incorporated into routine oral care activities and is typically performed by nursing staff or designated caregivers. To minimize burden, responsibility is assigned to a small number of staff members, and intraoral image capture is limited to triggered situations or to a limited periodic schedule for residents at higher baseline risk. AI-assisted processing can estimate intake and summarize component-level measurements without requiring manual calculation. Operational alerts are generated when these measurements, weight trends, EHR context, or nurse concern meet locally defined operational thresholds. This approach is intended to limit unnecessary alerts while preserving clinical review for residents whose intake or oral status appears to be changing. Alert volume should be monitored during the pilot rather than assumed to be acceptable in advance. Pilot teams should predefine adjustment rules before rollout. For example, expansion should be delayed or paused if alert volume, missed assessments, delayed assessments, or documentation time exceed locally acceptable limits. Possible adjustments include narrowing eligibility to higher-risk residents, revising operational thresholds, adding protected time, redistributing tasks across shifts, simplifying documentation templates, or extending the pilot before scale-up.

The 24- to 48-hour bedside assessment is intended to support timely responses to identified risks, but it does require incremental nursing capacity. Some alerts will lead to additional bedside assessments, documentation, referrals, or care-plan changes. The purpose of the pilot is therefore not to assume that the workload is negligible, but to measure whether the alert volume, time per assessment, missed or delayed assessments, and documentation burden are feasible within existing staffing or require workflow redesign, threshold adjustment, narrower eligibility criteria, or additional protected time.

Documentation is primarily integrated into the EHR to avoid duplicate data entry. Some indicators (eg, action rate) are not part of routine frontline tasks and are instead assessed as part of periodic quality improvement activities. In practice, this review is conducted at defined intervals (eg, weekly during pilot-phase meetings or monthly service reviews) by LTC administrators or deputy managers responsible for service oversight. Because the review is limited to selected indicators and performed at these scheduled time points, it is intended to remain a manageable component of pilot implementation rather than a continuous additional burden. To support explainability in routine practice, nursing staff should provide brief, nontechnical explanations at the time of assessment or care planning, for example, by stating that the system noticed recent changes in how much the resident had been eating, or changes in oral appearance compared with the resident’s usual pattern, and that these changes prompted a follow-up check. The goal is to explain why the review is occurring in resident- and proxy-facing language, rather than to present technical details of the model.

### Hypothetical Case Vignette

Consider a hypothetical 78-year-old woman with moderate dementia and ill-fitting dentures who begins to leave soft foods unfinished. Over 5 days, routine meal-tray images suggest that her estimated intake has fallen by approximately 30% compared with her 2-week baseline [1]. Rather than displaying only a global risk label, the staff-facing alert states that intake has declined substantially from her usual pattern and recommends bedside nutrition review. The nutrition champion receives the alert at 9 AM and arranges a bedside assessment before the following day. Because the resident also appears uncomfortable while chewing, a selective intraoral image is obtained during oral care. The image-based tool flags possible gingival inflammation within its validated scope [2], and nurse assessment confirms denture discomfort and oral pain. A ServiceRequest triggers denture review and a dietitian consult. Interim measures include fortified soups and analgesic oral gel. Within 72 hours, the dentures are adjusted; plate-waste metrics improve and body weight stabilizes over 2 weeks. Each step is documented through EHR write-back so that subsequent shifts can see what was done [14,16]. The vignette illustrates how component-level AI outputs, nurse verification, and operational thresholds can lead to action within the 48-hour window.

## Discussion

### Key Message

AI-enabled clinical monitoring for oral and nutrition risk improves care only when it is embedded in nursing policy and routines. The framework presented here makes that idea concrete by specifying accountable roles, a time-bound response, explicit integration steps, and measurable KPIs with scheduled threshold tuning. It is conceptually informed by key implementation outcomes—adoption, fidelity, and sustainability—proposed by Proctor et al [16]. The playbook proposes a conservative route to start small, monitor burden, and iterate without losing sight of resident dignity and consent. In effect, the paper translates promising accuracy reports from intake tracking and oral screening [1,2,7,8] into day-to-day practices by specifying how AI-derived measurements, operational thresholds, nurse verification, and ethical safeguards can be combined in routine care [9,12,20]. This distinction is important because not every AI component in the workflow is a risk-prediction model; some tools provide measurements that must still be interpreted through clinical context and nursing judgment. Although the proposed pilot is described here as an implementation pathway rather than a formal trial protocol, future prospective evaluations of the bundle should be designed and reported using established frameworks appropriate to study design, such as SPIRIT-AI (Standard Protocol Items: Recommendations for Interventional Trials-Artificial Intelligence) for protocol development, CONSORT-AI (Consolidated Standards of Reporting Trials-Artificial Intelligence) for interventional evaluation, or SQUIRE 2.0 (Standards for Quality Improvement Reporting Excellence 2.0) for quality-improvement reporting, to support transparency, reproducibility, and rigorous assessment.

### Comparison With Prior Work

Existing reviews and feasibility studies describe the burden of oral disease and malnutrition in nursing homes and the limitations of current instruments [3-6], and report encouraging performance for AI tools that quantify intake or screen for oral inflammation [1,2,7,8]. Informatics studies remind us that decision support must be trustworthy, calibrated, and aligned to workflow, and they show that SMART on FHIR allows sustainable integration without one-off interfaces [14,15]. The contribution here is to connect those strands from a nursing perspective and to specify policy levers—champion roles with protected time, a 48-hour response rule, standards-based write-back, and auditable KPIs—that suit resource-constrained facilities and are inspectable by managers and regulators. The approach is consistent with calls for digital readiness in nursing [10,11,19] and with guidance on equitable, responsible AI in oral health [12].

### Regulation, Accountability, and Vendor Due Diligence

Depending on jurisdiction, tools that estimate intake or screen for oral lesions may qualify as Software as a Medical Device (SaMD). In such cases, good machine learning practice, postmarket monitoring, and change management become relevant for deployers and developers [23,24]. Under the European AI Act, many clinical AI applications are likely to fall within high-risk use contexts, with corresponding expectations regarding risk management, technical documentation, postdeployment oversight, and human review [25]. Facility-level KPI tuning should therefore be understood as one component of operational oversight and local performance monitoring, not as a substitute for manufacturer quality-management obligations or formal postmarket surveillance processes where these apply. Facilities should therefore verify how vendors manage model updates, track performance under dataset shift, such as seasonal lighting changes in dining rooms, and maintain audit trails that link model versions and thresholds to human actions using EHR provenance and audit resources. Across jurisdictions, nursing leadership is increasingly integrated into facility governance to coordinate model oversight, maintain documentation standards, and contribute to safety reporting for AI-supported assessments. Clear allocation of responsibility remains essential, with human review of high-priority outputs, documented escalation, and transparent communication with residents and proxies.

### Limitations

This viewpoint synthesizes emerging evidence and proposes a pragmatic framework, but several limitations warrant careful consideration. First, as a conceptual paper without original data, it cannot establish cost-effectiveness or net clinical benefit, and the 48-hour response standard, champion roles, alert-volume range, and KPI thresholds remain design hypotheses not yet prospectively tested as a bundle. The proposed 0.2 to 0.4 FTE estimate and 48-hour rule are author-proposed planning assumptions informed by clinical and implementation experience, rather than figures derived from formal consensus, historical workload data, or theoretical modeling. Second, the empirical base is modest and only partly generalizable: several cited studies are small, single-country, or conducted outside residential LTC, and accuracy may vary with diet patterns, lighting, denture status, and cognitive impairment [1,2,7,8]. Third, measurement reliability and model performance can degrade in practice: KPI estimates depend on consistent documentation, accurate time-stamping, and clean data flows (with action rates susceptible to inflation by low-value interventions and alert-to-assessment intervals misestimated if tasks close late), and model accuracy may drift with changes in lighting, camera angles, resident mix, or menus despite scheduled tuning. Fourth, integration and market constraints may impede implementation: even with SMART on FHIR, interoperability requires effort (version mismatches, terminology mapping, and security reviews), some EHRs limit write-back, and proprietary tools may lack transparency about model versions, data provenance, or update cadence, complicating replication and independent benchmarking [14]. Fifth, regulatory heterogeneity and ethical-sociobehavioral risks persist: jurisdictions differ in SaMD classification, update governance, and reimbursement, potentially widening gaps between well-resourced and underresourced facilities [21-23], and continuous monitoring raises oversurveillance concerns in cognitively impaired populations; while safeguards (layered consent, minimum-necessary capture, opt-out recording, and subgroup monitoring) are proposed, their effectiveness depends on local governance and training and may not fully prevent behavioral effects that bias the data [9,12,20]. Even within these limitations, the framework’s value lies in translating fragmented pilot efforts into a coherent policy direction for nursing-led AI oversight. Finally, validation of the proposed framework across multiple sites and jurisdictions remains necessary. Future multicenter studies should examine the feasibility, workflow integration, and performance of the framework across diverse LTC settings, including variations in staffing models, regulatory environments, and care processes. Implementation fidelity during data capture is also uncertain. Meal-image capture may be disrupted by shared food, spills, tray clearing in batches, poor lighting, resident refusal, or difficulty linking images to the correct resident. Selective intraoral imaging may be limited by discomfort, cognitive impairment, lighting, and staff skill. Future pilots should therefore report capture completion, image quality, missed-capture reasons, linkage errors, refusal or opt-out rates, and staff time, rather than reporting only downstream clinical KPIs.

### Conclusions

Continuous, objective clinical monitoring of eating and oral health is increasingly technically feasible. What has been missing are clear roles, timelines, and accountability. A nursing-led framework that couples AI-derived measurements and operational alerts to timely bedside assessment and documented follow-up—supported by standards-based EHR integration [14], ethical guardrails [9,12,20], and prevention-oriented incentives—offers a practical route from pilot projects to routine practice. Co-designed implementation studies that report standardized outcomes will accelerate learning, inform reimbursement, and help ensure reliable, equitable deployment in LTC [16].

## Abbreviations

CONSORT-AI: Consolidated Standards of Reporting Trials-Artificial Intelligence
EHR: electronic health record
FHIR: Fast Healthcare Interoperability Resources
KPI: key performance indicator
LTC: long-term care
SaMD: Software as a Medical Device
SMART: Substitutable Medical Applications and Reusable Technologies
SPIRIT-AI: Standard Protocol Items: Recommendations for Interventional Trials-Artificial Intelligence
SQUIRE 2.0: Standards for Quality Improvement Reporting Excellence 2.0
